# Supplementary material for: Intestinal FGF15/19 physiologically repress hepatic lipogenesis in the late fed-state by activating SHP and DNMT3A
Source: Nat Commun. 2020 Nov 24;11:5969. doi: 10.1038/s41467-020-19803-9 (PMC7686350; doi:10.1038/s41467-020-19803-9)
Supplement: Supplementary file 1 — Supplementary Information [file 41467_2020_19803_MOESM1_ESM.pdf]

Supplementary Information

Intestinal FGF15/19 physiologically repress hepatic lipogenesis in the late fed-state by activating SHP and DNMT3A

Kim *et al.*

Supplementary Figures

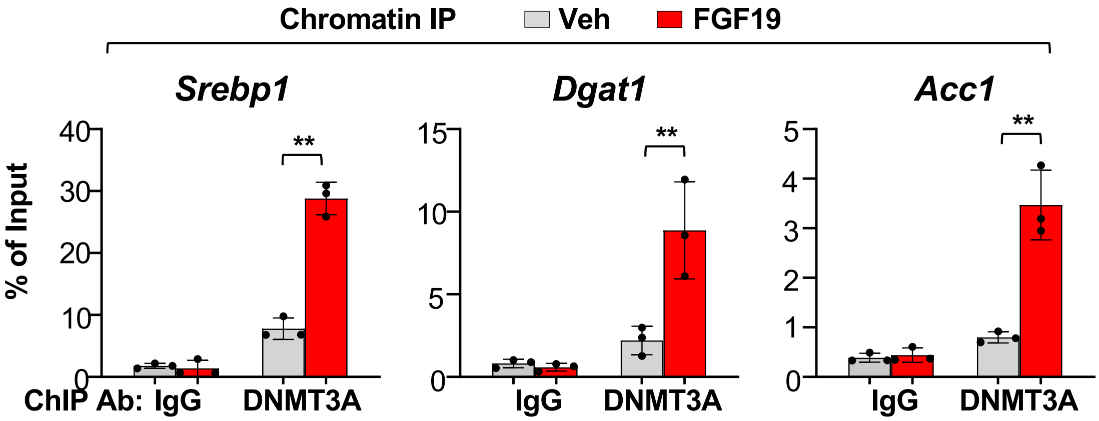

Supplementary Figure 1. FGF19 treatment increases DNMT3A occupancy at lipogenic gene promoters. C57BL/6 mice were treated with vehicle or FGF19 for 2 h after fasting overnight. Occupancy of DNMT3A at *Srebp1*, *Dgat1*, and *Acc1* determined by mouse liver ChIP assay. Mean and standard deviation are plotted (n=3 mice). Statistical significance was determined by two-way ANOVA with the Tukey posttest. $^{**}P < 0.01$.

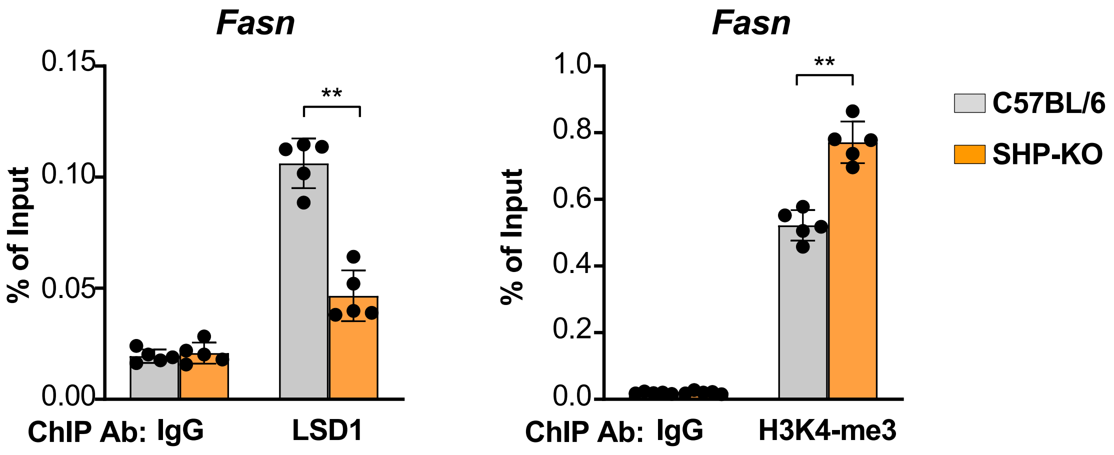

Supplementary Figure 2. LSD1 binding is decreased and histone H3K4-me3 levels are increased at the *Fasn* promoter in livers of SHP-KO compared to control mice. Occupancy of LSD1 and H3K4-me3 levels at the *Fasn* promoter was examined by ChIP assays in livers of C57BL/6 mice and SHP-KO mice. Mean and standard deviation are plotted (n=5 mice). Statistical significance was determined by two-way ANOVA with the Tukey posttest. **P < 0.01.

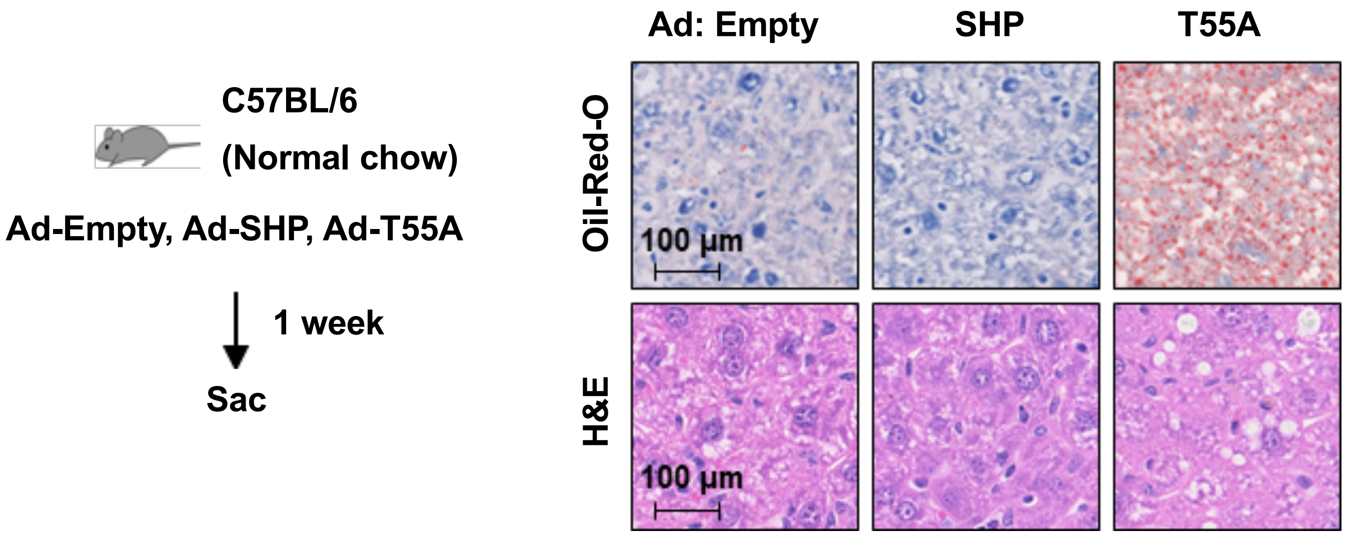

Supplementary Figure 3. Effects of adenoviral-mediated expression of SHP-WT and T55A mutant on hepatic lipid levels in mice. C57BL/6 mice were infected with adenovirus expressing wild type-SHP or T55A-SHP, a phosphorylation-defective mutant, or empty adenovirus, and one weeks later, the mice were sacrificed. Experimental outline (left) and images of liver sections (right) stained with H&E (bottom) or Oil Red O (top). Representative images are shown for one mouse in each group. Similar results were observed in two additional mice in each group.

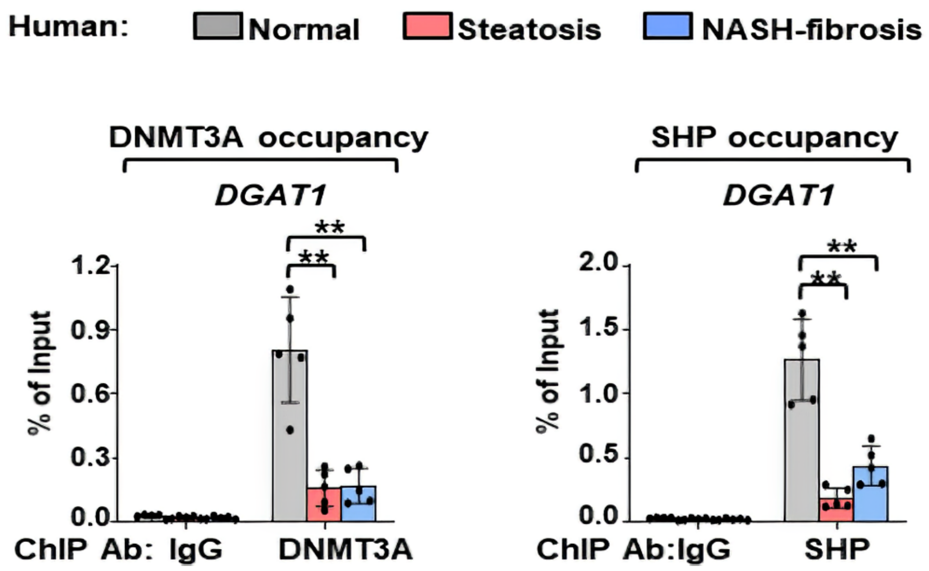

Supplementary Figure 4. In NAFLD patients, occupancy of DNMT3A and SHP at the *DGAT1* gene promoter is decreased. Liver extracts from 15 individuals were randomly combined to form 5 pooled samples each containing 3 individual samples. DNMT3A (left) and SHP (right) occupancies at the *DGAT1* promoter determined by ChIP. The mean and standard deviation are plotted. (n = 5 individuals) Statistical significance was determined by two-way ANOVA with the Tukey posttest. $^{**}P < 0.01$.

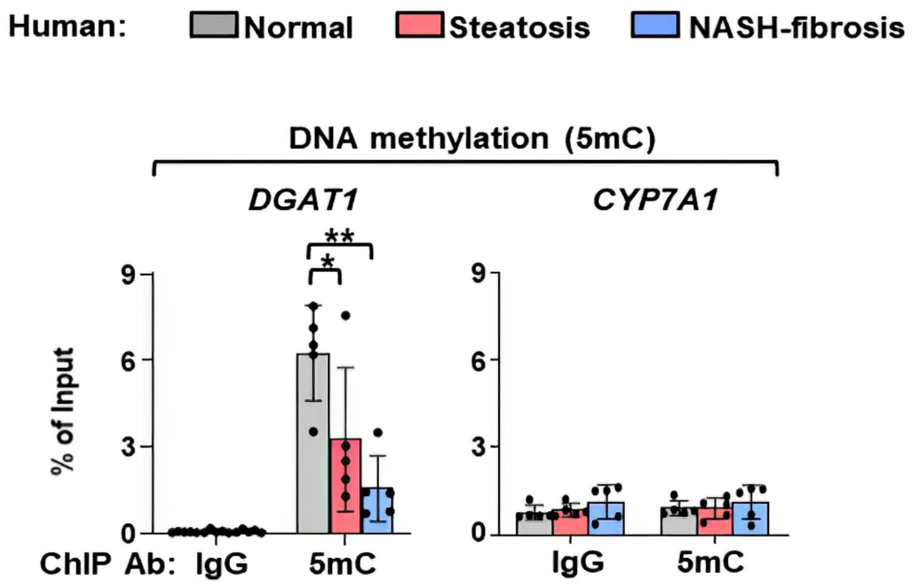

Supplementary Figure 5. DNA methylation at the *DGAT1* gene promoter is decreased in livers of NAFLD patients. Methylated DNA at *DGAT1* promoter and *CYP7A1* determined by MeDIP assay. The mean and standard deviation are plotted. (n = 5 individuals) Statistical significance was determined by two-way ANOVA with the Tukey posttest. *P < 0.05, **P < 0.01.

Supplementary Tables

Supplementary Table 1. Antibody Information

Antibody	Company	# of catalogue	Species	Reactivity	Dilutions
SHP (H-160)	Santa Cruz Biotechnology	sc-30169	rabbit polyclonal IgG	human and mouse	1:3000 for IB
DNMT3A (D-15)	Santa Cruz Biotechnology	sc-10232	goat polyclonal IgG	human and mouse	1:3000 for IB
DNMT3B (T-16)	Santa Cruz Biotechnology	sc-10236	goat polyclonal IgG	human and mouse	1:3000 for IB
DNMT1 (C-17)	Santa Cruz Biotechnology	sc-10222	goat polyclonal IgG	human and mouse	1:3000 for IB
SREBP1 (H-160)	Santa Cruz Biotechnology	sc-8984	rabbit polyclonal IgG	human and mouse	1:3000 for IB
β-Tubulin (H-235)	Santa Cruz Biotechnology	sc-9104	rabbit polyclonal IgG	human and mouse	1:5000 for IB
Lamin A (H-102)	Santa Cruz Biotechnology	sc-20680	rabbit polyclonal IgG	human and mouse	1:5000 for IB
FASN (C20G5)	Cell Signaling	3180	rabbit IgG	human and mouse	1:5000 for IB
β-Actin(13E5)	Cell Signaling	4970	rabbit IgG	human and mouse	1:10000 for IB
5-methylcytosine (clone 7D21)	Zymo Research	A3001-200	mouse IgG	human and mouse	No dilution for MeDIP

Supplementary Table 2. Primer sequences

a. List of mouse primer sequences

**pre-mRNA**		
36b4	F	CGTAACCAGCAAGGAAGCTG
36b4	R	TGTCTGCTCCCACAATGAAG
Fasn	F	CTCCTACAGGGTTGGCTCTG
Fasn	R	CCAGACCGCTTGGGTAATC
Srebp1	F	GGCCACTCTTCTTCCATCAC
Srebp1	R	TGCCACTCACCATCCTACAG
Acc1	F	CTCCAGCAGAATTTGTTACTCG
Acc1	R	ACATGCTAGGGAGACAGAAAGG
**mRNA**		
36b4	F	GATTCGGGATATGCTGTTGG
36b4	R	AAGCCTGGAAGAAGGAGGTC
Fasn	F	CCAAGTACCATGGCAACGTG
Fasn	R	AGCCAGGGAGCTATGGATGA
Srebp1	F	TGCTCCAGCTCATCAACAAC
Srebp1	R	AGAGAGGAGGCCAGAGAAGC
Acc1	F	TGAGGAGGACCGCATTTATC
Acc1	R	CATGGGATGGCAGTAAGGTC
Acly	F	TGTTGACATTGGAGCCCTC
Acly	R	GATACAGCCCTTGCTTCAG
Dgat1	F	GTCAAGGCCAAAGCTGTCTC
Dgat1	R	CGGTAGGTCAGGTTGTCTGG
Lipin	F	TCCTCTACTTCTGGCGATGC
Lipin	R	TGTGGCATTTCTTCCTCTCC
Cyp7a1	F	CAGCATCCTCTTGCTACTTGG
Cyp7a1	R	TGAGGCTGTCTGTGATGTCC
**ChIP / MeDIP**		
Fasn	F	TAGGCAATAGGGTGATGGG
Fasn	R	ATCCTGGTCTCCAAGGTG
Srebp1	F	GTGTCCAGTTCGCACATCTC
Srebp1	R	CAGTCTCAACCCGCTAGGC
Cyp7a1	F	ACCTTCGGCTTATCGACTATTGC
Cyp7a1	R	TATCTGGCCTTGAACTAAGTCCATCT
Esr1	F	ACAGTGGGCTTGCTGTTGTC
Esr1	R	ACCAGATCCAAGGGAACGAG

b. List of human primer sequences

**mRNA**		
FASN	F	GTGTCCACCAGCAACATCAG
FASN	R	GTTCTCCAGCAAGCCATCTC
SREBP1	F	GTCTTCCTCTGCCTGTCCTG
SREBP1	R	GACGCTGGTGGTATCTGAGG
DGAT1	F	CTCAGTGCAGGATGGTCAAC
DGAT1	R	ACCAGACCCACACCAGAAAG
DNMT3A	F	GGACAAGAATGCCACCAAAG
DNMT3A	R	CATCCACCAAGACACAATGC
SHP	F	CAGAGATCAGGTGGGCAGAG
SHP	R	TGTGGCTGAGTGAAGAGCTG
**ChIP**		
FASN	F	GGCTGTTGGTGGCTTTCC
FASN	R	CTCCTCATCCTCCGCTCTC
SREBP1	F	GAGACAAAGGCCAGGGAGAC
SREBP1	R	CTGACCGACATCGAAGGTG
