# Supplementary material for: Intestinal FGF15/19 physiologically repress hepatic lipogenesis in the late fed-state by activating SHP and DNMT3A
Source: Nat Commun. 2020 Nov 24;11:5969. doi: 10.1038/s41467-020-19803-9 (PMC7686350; doi:10.1038/s41467-020-19803-9)
Supplement: Supplementary file 3 — Reporting Summary [file 41467_2020_19803_MOESM3_ESM.pdf]

nature research

Corresponding author(s): Jongsook Kim Kemper

Last updated by author(s): Aug 27, 2020

# Reporting Summary

Nature Research wishes to improve the reproducibility of the work that we publish. This form provides structure for consistency and transparency in reporting. For further information on Nature Research policies, see our Editorial Policies and the Editorial Policy Checklist.

## Statistics

For all statistical analyses, confirm that the following items are present in the figure legend, table legend, main text, or Methods section.

| n/a | Confirmed | |
|---|---|---|
| ☐ | ☒ | The exact sample size (*n*) for each experimental group/condition, given as a discrete number and unit of measurement |
| ☐ | ☒ | A statement on whether measurements were taken from distinct samples or whether the same sample was measured repeatedly |
| ☐ | ☒ | The statistical test(s) used AND whether they are one- or two-sided<br>*Only common tests should be described solely by name; describe more complex techniques in the Methods section.* |
| ☒ | ☐ | A description of all covariates tested |
| ☐ | ☒ | A description of any assumptions or corrections, such as tests of normality and adjustment for multiple comparisons |
| ☐ | ☒ | A full description of the statistical parameters including central tendency (e.g. means) or other basic estimates (e.g. regression coefficient) AND variation (e.g. standard deviation) or associated estimates of uncertainty (e.g. confidence intervals) |
| ☒ | ☐ | For null hypothesis testing, the test statistic (e.g. *F*, *t*, *r*) with confidence intervals, effect sizes, degrees of freedom and *P* value noted<br>*Give P values as exact values whenever suitable.* |
| ☒ | ☐ | For Bayesian analysis, information on the choice of priors and Markov chain Monte Carlo settings |
| ☒ | ☐ | For hierarchical and complex designs, identification of the appropriate level for tests and full reporting of outcomes |
| ☒ | ☐ | Estimates of effect sizes (e.g. Cohen's *d*, Pearson's *r*), indicating how they were calculated |

*Our web collection on statistics for biologists contains articles on many of the points above.*

## Software and code

Policy information about availability of computer code

| | |
|---|---|
| Data collection | No software was used for data collection |
| Data analysis | GraphPad Prism 6 (GraphPad software version 6.01) was used for data analysis. RNA-seq: Sequencing alignment was performed by STAR ver 2.5.0a. The project-specific parameters used were '-type histone -species mm10 –use_pooled_ctl'. Gene ontology analysis was performed using the program DAVID. |

For manuscripts utilizing custom algorithms or software that are central to the research but not yet described in published literature, software must be made available to editors and reviewers. We strongly encourage code deposition in a community repository (e.g. GitHub). See the Nature Research guidelines for submitting code & software for further information.

## Data

Policy information about availability of data

All manuscripts must include a data availability statement. This statement should provide the following information, where applicable:

- Accession codes, unique identifiers, or web links for publicly available datasets
- A list of figures that have associated raw data
- A description of any restrictions on data availability

The RNA-seq in the present study and the published SHP ChIP-seq data 19 are deposited in the Gene Expression Omnibus (GEO) database with the Accession Numbers ____ and GSE74913, respectively.

# Field-specific reporting

Please select the one below that is the best fit for your research. If you are not sure, read the appropriate sections before making your selection.

☒ Life sciences ☐ Behavioural & social sciences ☐ Ecological, evolutionary & environmental sciences

For a reference copy of the document with all sections, see nature.com/documents/nr-reporting-summary-flat.pdf

# Life sciences study design

All studies must disclose on these points even when the disclosure is negative.

| | |
|---|---|
| Sample size | In general a minimum of three independent biological samples or mice were used. Usually up to 5 mice were independently studied due to increased variability in in vivo studies. |
| Data exclusions | No data was excluded |
| Replication | Experiments were repeated at least two times to ensure the results could be replicated |
| Randomizatio | Animals were arbitrarily randomly assigned to groups, but no specific method of randomization was used |
| Blinding | Investigators were not blinded to the sample groups. |

# Reporting for specific materials, systems and methods

We require information from authors about some types of materials, experimental systems and methods used in many studies. Here, indicate whether each material, system or method listed is relevant to your study. If you are not sure if a list item applies to your research, read the appropriate section before selecting a response.

## Materials & experimental systems

| n/a | Involved in the study | |
|---|---|---|
| ☐ | ☒ | Antibodies |
| ☐ | ☒ | Eukaryotic cell lines |
| ☒ | ☐ | Palaeontology and archaeology |
| ☐ | ☒ | Animals and other organisms |
| ☒ | ☐ | Human research participant |
| ☒ | ☐ | Clinical data |
| ☒ | ☐ | Dual use research of concern |

## Methods

| n/a | Involved in the study | |
|---|---|---|
| ☒ | ☐ | ChIP-seq |
| ☒ | ☐ | Flow cytometry |
| ☒ | ☐ | MRI-based neuroimaging |

## Antibodies

| | |
|---|---|
| Antibodies used | Antibodies are described in Methods - first paragraph. |
| Validation | Commercial antibodies were used that were validated by the manufacturer. |

## Eukaryotic cell lines

Policy information about cell lines

| | |
|---|---|
| Cell line source(s) | ATCC |
| Authentication | Nine |
| Mycoplasma contamination | Not tested for Mycoplasma contaimination |
| Commonly misidentified lines (See ICLAC register) | N/A |

## Animals and other organisms

Policy information about studies involving animals; ARRIVE guidelines recommended for reporting animal research

| | |
|---|---|
| Laboratory animals | Animal information is reported in Methods. |

| | |
|---|---|
| Wild animals | None |
| Field-collected samples | None |
| Ethics oversight | ACUC and Institutional Biosafety Committees of the University of Illinois at Urbana-Champaign |

Note that full information on the approval of the study protocol must also be provided in the manuscript.
